# *Campylobacter fetus* Bacteremia Revealed by Cellulitis without Gastrointestinal Symptoms in the Context of Acquired Hypogammaglobulinemia: A Report of Three Cases

**DOI:** 10.1155/2011/628902

**Published:** 2011-07-09

**Authors:** Souleymane Brah, Laurent Chiche, Marion Brun, Nicolas Schleinitz, Jean-Robert Harle, Jean-Marc Durand

**Affiliations:** Service de Médecine Interne, Hôpital de la Conception, 147 Bd Baille, 13005 Marseille, France

## Abstract

*Campylobacter fetus* bacteremia is rare and occurs mainly in patients with immunosuppression. This infection, which often involves secondary localizations has already been reported in some primary humoral immune deficiencies. We describe three cases of severe infection due to *C. fetus* with cellulitis at presentation, but without any gastrointestinal symptoms, occurring in patients with acquired hypogammaglobulinemia.

## 1. Introduction

Infections due to *Campylobacter fetus* represent only a small proportion of *Campylobacter* infections, usually responsible for acute diarrhea. *C. fetus* bacteremia is relatively rare and occurs mainly against a background of immunosuppression (diabetes, cirrhosis, AIDS, and elderly patients) [1, 2]. This severe form of infection that can spread to involve secondary localizations has been reported in some patients with primary humoral immune deficiencies [3, 4]. 

We describe three cases of severe infection due to *C. fetus* occurring in immunosuppressed patients with acquired hypogammaglobulinemia.

## 2. Case Report

**Patient 1**

A 77-year-old diabetic man, with chronic lymphocytic leukemia (not treated), was hospitalized for sepsis without any obvious clinical source, notably no diarrhea syndrome. He received empiric antibiotherapy with a combination of ceftriaxone and metronidazole. Apart from known hyperlymphocytosis (24 giga/L), the rest of his hemogram was unremarkable. There was clear hypogammaglobulinemia: IgG = 3.2 g/L (normal >6.9 g/L) and IgA = 0.4 g/L (normal >0.88 g/L). *C. fetus* was isolated from blood cultures, but not from the fecal culture. Azithromycin was initiated for a total duration of 2 weeks. One month later, the patient presented with a novel septic state, this time associated with abdominal cutaneous cellulitis. *C. fetus* was again recovered from blood cultures (5/5 bottles). Initial antibiotherapy (piperacillin-tazobactam) was again replaced by azithromycin for a duration of 4 weeks. Substitution treatment with monthly intravenous immunoglobulins (IVIg) was initiated, and a residual level of 6 g/L was obtained. No relapse was noted after more than 12 months of followup.

**Patient 2**

A 58-year-old man with SHARP syndrome (mixed connective tissue disease with predominant autoimmune myositis) and hypogammaglobulinemia secondary to immunosuppressive treatment (cyclophosphamide, mycophenolate mofetil, and rituximab) was hospitalized for sepsis associated with cellulitis of the left upper arm, without any obvious clinical source. His hemogram was unremarkable (neutrophils = 16 giga/L). The patient had already received monthly IVIg substitution for 4 years and had an IgG level of 6 g/L and IgA of 1.05 g/L at the time of appearance of the infection. Blood cultures (5/5 bottles) were positive for *C. fetus*. The infection responded rapidly to combined antibiotherapy (piperacillin-tazobactam and azithromycin) for a duration of 6 weeks. No relapse was noted after a followup of 19 months.

**Patient 3**

A 71-year-old woman, followed for several years with mixed cryoglobulinemia complicating low-grade lymphoma, was hospitalized for sepsis at 1 month after her first course of chemotherapy (rituximab-cyclophosphamide and dexamethasone). The chemotherapy was rapidly followed by the development of infectious complications including pneumopathy and then otitis due to Pneumococcus. Severe hypogammaglobulinemia (IgG = 2.89 g/L and IgA = 0.64 g/L) led to substitution treatment with IVIg. One month later, in the absence of neutropenia, she presented with cellulitis of the left leg with reactional arthritis of the homolateral knee (IgG = 5.25 g/L at that time). Blood cultures were positive for *C. fetus* (2/2 bottles). She was successfully treated with ceftriaxone 2 g/day followed by oral ciprofloxacin (1000 mg/day) for a total duration of 3 weeks. Treatment with IVIg was continued with the aim of achieving a residual level of >6 g/L, but at 2 months she presented with a new episode of severe sepsis due to *Escherichia coli* leading to death.

## 3. Discussion 

We report three cases of severe *C. fetus* bacteremia developing in the setting of acquired immunosuppression with hypogammaglobulinemia. These “opportunistic” infections are a real concern for the clinician since *Campylobacter* bacteremia often involves extraintestinal localizations [1, 2, 5]. *C. fetus*, which is not the most common *Campylobacter* species identified in the general population, is more often found in immunosuppressed patients where it represents more than one-half of cases [2]. As in our patients, this species also appears to be more common in elderly patients, as reported by Pacanowski et al. (median age 69.5 years versus 55.6 for other species of *Campylobacter*) [2]. 

Since the introduction of tritherapy, the number of cases reported in connection with HIV has been superseded by those occurring in other types of immunosuppression, particularly in subjects with humoral immune deficiencies. The first case reported in the literature concerned a 50-year-old patient with Good's syndrome who presented with bacteremia due to *C. fetus* postthymectomy [1]. Other studies then demonstrated the association between hypogammaglobulinemia and infection with different *Campylobacter* species [2]. More recently, Oksenhendler et al. found 19 cases of common variable immunodeficiency (CVID) in a series of 252 *Campylobacter* infections, and measurement of immunoglobulins in these patients revealed undetectable levels of IgA and IgM [4]. This hypogammaglobulinemia, which usually affects IgA, could favor *C. fetus* infections due to the important role of these immunoglobulins in the anti-infectious defense of the body at the level of the digestive mucous membranes. 

The digestive tract is the usual route of contamination by *C. fetus*, yet digestive symptoms such as diarrhea are not always present (less than one patient in two). This may divert the clinician away from the diagnosis and explains in part why the diagnosis is most often made from blood cultures and not from fecal cultures [2, 5]. This absence of digestive signs associated with atypical symptoms such as cellulitis are characteristics of *C. fetus* infection reported in the literature [2, 5]. In the cohort reported by Pacanowski et al., diarrhea was found in only 33% of cases and cellulitis in 19% (versus 7% cellulitis for the other *Campylobacter* species) [2]. These data agree with the clinical presentation in our three patients who all presented with cellulitis-type cutaneous symptoms, without any digestive signs. 

The evolution of the patients in terms of mortality depends on the underlying disease and also on the initial choice (adapted or not) of antibiotherapy. *C. fetus* is often resistant to quinolones and macrolides, and some authors recommend the use of imipenems as first-line treatment [1, 2]. In the series of 178 patients described by Pacanowski et al., no strain of *C. fetus* was resistant to imipenems, while 32% were resistant to fluoroquinolones, and no patient on imipenem had died at 30-day followup [2]. In contrast, resistance to erythromycin and the combination amoxicillin-clavulanic acid was more often found with other *Campylobacter* species [2]. 

Bacteremia due to *C. fetus* readily recurs with atypical symptoms and requires prolonged antibiotherapy [2, 5]. Multiple factors, including the duration of antibiotherapy, probably favor these relapses, but hypogammaglobulinemia could also be a risk factor for relapse even in cases of prolonged antibiotherapy [1]. Some studies have demonstrated that resistance of the bacteria is due to a defect in opsonization. The impact of substitution treatment with IVIg is poorly understood, especially as the IgA deficit is not corrected by this therapy. 

Our three cases illustrate both the atypical presentation with absence of gastrointestinal symptoms and the potential risk of severe *C. fetus* infections in the context of hematological or rheumatologic conditions with acquired hypogammaglobulinemia. Actually, *C. Fetus* bacteremia should be suspected among any immunocompromised patients with gastrointestinal symptoms, as well as among those presenting with cellulitis but no diarrhea.
